# Terminology in morphological anomalies of the cerebellum does matter

**DOI:** 10.1186/s40673-015-0027-x

**Published:** 2015-07-07

**Authors:** Andrea Poretti, Eugen Boltshauser

**Affiliations:** Section of Pediatric Neuroradiology, Division of Pediatric Radiology, Russell H. Morgan Department of Radiology and Radiological Science, The Johns Hopkins University School of Medicine, 1800 Orleans Street, Baltimore, MD 21287 USA; Department of Pediatric Neurology, University Children’s Hospital, Zurich, Switzerland

**Keywords:** Cerebellum, Neuroimaging, Cerebellar atrophy, Cerebellar hypoplasia, Cerebellar dysplasia, Cerebellar dysmorphia

## Abstract

Neuroimaging plays a key role in the diagnostic work-up of morphological abnormalities of the cerebellum. Diagnostic criteria for numerous morphological anomalies of the cerebellum are based on neuroimaging findings. Various morphological patterns have been described on neuroimaging including cerebellar hypoplasia, cerebellar agenesis, pontocerebellar hypoplasia, cerebellar dysplasia, cerebellar dysmorphia, and cerebellar atrophy. These patterns have specific differential diagnoses. The familiarity with the diagnostic criteria is mandatory for a correct diagnosis and a targeted work-up to avoid unnecessary investigations. A correct diagnosis is essential for early therapy, prognosis, and counseling of the affected children and their family.

## Background

Progress in neuroimaging techniques and genetic analysis has led to a significant improvement in definition of morphological abnormalities of the cerebellum. New classifications and checklists of morphological abnormalities of the cerebellum have been proposed [1–3].

Neuroimaging plays a key role in the diagnostic work-up of morphological abnormalities of the cerebellum [4, 5]. Diagnostic criteria for numerous morphological anomalies of the cerebellum are based on neuroimaging findings. Various morphological patterns have been described on neuroimaging including cerebellar hypoplasia, cerebellar agenesis, pontocerebellar hypoplasia, cerebellar dysplasia, cerebellar dysmorphia, and cerebellar atrophy. These patterns have specific differential diagnoses. The familiarity with the diagnostic criteria is mandatory for a correct diagnosis and a targeted work-up to avoid unnecessary investigations. An accurate diagnosis of these complex abnormalities is important for: 1) early institution of the correct therapy, 2) prediction of the prognosis, and 3) counseling of the family including inheritance pattern and risk of recurrence.

Sometimes in the daily clinical work as well as in the scientific literature, the terms mentioned above are used interchangeably. This leads to confusion and may result in misdiagnosis. Semantics does matter in medical and scientific communications [6]. Precision in the use of language, whether verbal or written, is a reflection of precision in scientific thought and patient care.

Here, we will 1) differentiate between cerebellar malformations and cerebellar disruptions and 2) define and discuss various morphological cerebellar patterns including cerebellar agenesis, cerebellar hypoplasia, pontocerebellar hypoplasia, cerebellar dysplasia, cerebellar dysmorphia, and cerebellar atrophy.

### Cerebellar malformations versus cerebellar disruptions

The diagnosis of congenital cerebellar anomalies should include the differentiation between inherited (developmental) and acquired (disruptive) abnormalities. A malformation is defined as a congenital morphologic anomaly of a single organ or body part due to cellular and molecular pathways involved in organogenesis; the molecules in these pathways can be altered by gene mutations, teratogens, or combined effects [7]. A disruption is defined as a congenital morphologic anomaly due to the breakdown of a body structure that had a normal developmental potential [7]. Disruptions may be caused by e.g. prenatal infection, hemorrhage, or ischemia and commonly involve the cerebellum [2]. Disruptions are acquired lesions with very low recurrence risk. However, a genetic predisposition to disruptive lesions may be present. Dominant mutations in *COL4A1* lead to change of the basal membrane of capillaries resulting in microangiopathy [8]. Within the brain, the microangiopathy may lead to hemorrhage and/or ischemia and result in a spectrum of lesions including porencephaly or unilateral cerebellar hypoplasia [9, 10].

### Cerebellar hypoplasia

**Fig. 1:**
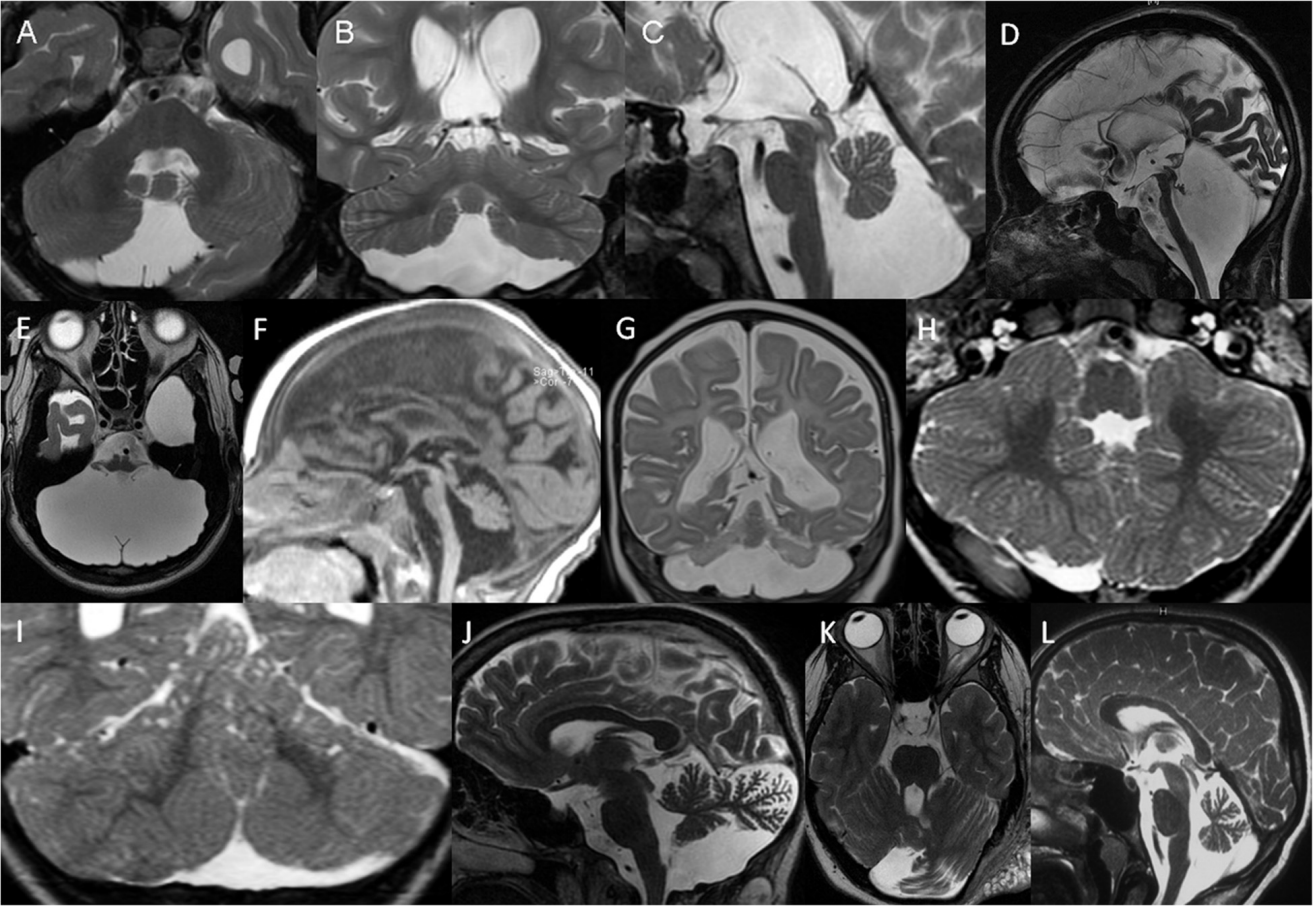
**a**, Axial, **b**, Coronal, and **c**, Sagittal T2-weighted images of a 17-year-old male with seizures and cerebellar hypoplasia of unknown origin show reduced volume, but near normal structure of the cerebellum. In addition, a cavum Vergae and reduced periventricular bilateral white matter are noted. **d** Sagittal and **e**, Axial T2-weighted images of a 15-year-old girl with cerebellar agenesis reveal almost complete absence of cerebellar tissue except for a rudimentary structure projecting posterior to the inferior colliculi and lateral to the brainstem. The pons is markedly hypoplastic and the posterior fossa is enlarged (reprinted with permission from Poretti A et al., Eur J Paediatric Neurol, 2009). **f** Sagittal T1- and **g** Axial T2-weighted images of a 1-year-old child with pontocerebellar hypoplasia type 2 and *TSEN54* mutations show marked hypoplasia and reduction in the size of the cerebellar hemispheres with relative preservation of the midline vermis, resulting in a characteristic “dragonfly” appearance (reprinted with permission from Bosemani T et al., Radiographics, 2015). **h** Axial and **i**, Coronal T2-weighted images of an 11-month-old child with Poretti-Boltshauser syndrome and *LAMA1* mutations reveal bilateral cerebellar dysplasia as abnormal cerebellar foliation, white matter arborization, and gray-white matter junction and multiple cortical/subcortical cysts located within the cerebellar vermis (mostly anterior and superior part) and both cerebellar hemispheres (mostly posterior and superior parts) (reprinted with permission from Poretti A et al., Cerebellum, 2014). **j** Sagittal and **k**, Axial T2-weighted images of 15-year-old patient with neurofibromatosis type 1 and cerebellar dysmorphia show enlargement of the left cerebellar hemisphere with enlarged interfolial spaces and bulky appearance of its posteromedial part, which crosses the midline. In addition, a plexiform neurofibroma is seen in the left soft tissue (reprinted with permission from Toelle SP et al., Cerebellum, 2015). **l** Sagittal T2-weighted image of a 5-year-old child with ataxia oculomotor apraxia type 1 disease and marked cerebellar atrophy as enlargement of the interfolial spaces

Cerebellar hypoplasia is a descriptive term implying a cerebellum with a reduced volume, but a normal shape and is stable over time (Fig. 1a-c) [11]. Cerebellar hypoplasia is a rather common finding and is associated with a highly heterogeneous group of diseases. Etiologies include prenatal infections and exposure to teratogens, chromosomal aberrations, metabolic disorders, genetic syndromes, and brain malformations. These include primary malformative and secondary disruptive lesions. The cerebellar involvement is heterogeneous: hypoplasia may affect the entire cerebellum (most commonly) or selectively involve the vermis alone or one/both hemispheres sparing the vermis.

Cerebellar hypoplasia may also present with enlarged cerebellar sulci (mimicking cerebellar atrophy), that is stable over time (in contrast to cerebellar atrophy). Because of the non-progressive course, we prefer the term hypoplasia to describe this imaging pattern [12–14].

Unilateral morphological anomalies of the cerebellum (e.g. unilateral cerebellar hypoplasia and unilateral cerebellar clefts) result from prenatal acquired injuries [15, 16]. In support of this concept, second trimester or early third trimester prenatal cerebellar hemorrhages have been shown by fetal MRI [17]. In addition, some patients have associated destructive lesions such as schizencephaly.

### Cerebellar agenesis

Cerebellar agenesis is defined by the near complete absence of cerebellar tissue with only remnants of the anterior vermis, flocculus, and/or middle cerebellar peduncles (Fig. 1d-e). A secondary pontine hypoplasia is typically seen. The definition of cerebellar agenesis is based on the morphologic pattern and does not suggest the pathogenesis [2]. Cerebellar agenesis may represent a malformation (e.g., mutations in *PTF1A*) [18] or a disruption (e.g., hemorrhage that occurs during gestation or in the perinatal period, vascular insufficiency in Chiari II malformation and cerebellar herniation, and as a sequela of prematurity) [2, 19].

### Pontocerebellar hypoplasia

The term pontocerebellar hypoplasia is often used in a descriptive manner to imply that the volume of the cerebellum and the pons is reduced. On the other hand, it is used specifically to refer to the pontocerebellar hypoplasias conceptualized by Peter Barth as early (prenatal) onset degenerative disorder (types 1, 2, 4, 5, 6, and 7) [20]. In this sense, the term “hypoplasia” is misleading and atrophy would be more accurate. In the last years, however, diseases with a non-progressive course have been included in the heterogeneous group of pontocerebellar hypoplasias (types 3 and 8). Listing in OMIM does not reflect pathogenesis.

Pontocerebellar hypoplasias as conceptualized by Peter Barth have a peculiar neuroimaging pattern including more pronounced involvement of the cerebellar hemispheres compared to the vermis and reduction in size of the pons (Fig. 1f-g) [21]. Predominant involvement of the cerebellar hemispheres is unusual and results in a “dragonfly” appearance on coronal images: flattened cerebellar hemispheres represent “the wings”, while the relatively preserved vermis represents “the body”. A “dragonfly” appearance has been shown also in very low birth weight (less than 1500 g) premature infants born before 32 weeks of gestation [22] and *CASK* mutations [23].

Reduction in size of the pons is not seen in the vast majority of diseases associated with cerebellar atrophy with postnatal onset [1]. Pontine hypoplasia however is a feature of diseases associated with prenatal loss of cerebellar tissue: 1) hereditary disorders with prenatal onset cerebellar atrophy including the group of pontocerebellar hypoplasias [21] and congenital disorder of glycosylation type 1a due to *PMM2* mutations [24], and 2) acquired disorders with prenatal onset cerebellar disruptive lesions such as cerebellar agenesis [2] and vanishing cerebellum in myelomeningocele [25], and anencephaly [26]. In addition, marked reduction in size of the pons has been shown in very low birth weight (less than 1500 g) premature infants born before 32 weeks of gestation [22, 27].

### Cerebellar dysplasia

Cerebellar dysplasia is defined by abnormal cerebellar foliation, white matter arborization, and gray-white matter junction (Fig. 1h). Dysplasia may globally involve the cerebellum or affect only one cerebellar hemisphere. In addition, cerebellar dysplasia may be associated with cortical/subcortical cysts [28]. Cerebellar cysts are likely the result of disturbed cortical migration/organization and pial membrane disruption and are most likely formed from the subarachnoid spaces that were engulfed by the dysplastic cerebellar folia, particularly in the boundary between the normal and dysplastic cerebellar cortex.

Global cerebellar dysplasia has been reported in a few posterior fossa malformations including Chudley-McCullough syndrome [29], α-dystroglycanopathies [30], *GPR56*-related polymicrogyria [31], and Poretti-Boltshauser syndrome due to *LAMA1* mutations [32, 33]. In α-dystroglycanopathies [30], *GPR56*-related polymicrogyria [31], and Poretti-Boltshauser syndrome due to *LAMA1* mutations [32, 33], cerebellar dysplasia is typically associated with cerebellar cysts. Diagonal folia across the vermis on axial images have been reported in tubulinopathies [34]. Dysplasia of the superior cerebellar vermis is generally seen in Joubert syndrome and can be very helpful when the other neuroimaging features (e.g. the molar tooth sign) are subtle or distorted [35]. Finally, cerebellar dysplasia may be the result of a disruptive process such as a prenatal hemorrhage. Disruptive cerebellar dysplasia may be uni- or bilateral and the affected hemispheres may be reduced in volume. In prenatal cerebellar disruptions, focal cerebellar dysplasia is typically confined to the region of the disrupted injury such as a cerebellar cleft [15]. In a few patients, disruptive cerebellar dysplasia may be associated with focal cerebellar cysts [28]. For specific diagnosis, it is important to determine the pattern of dysplasia and the presence of cerebellar cysts (Fig. 1i) and correlate them with clinical information [28].

Lhermitte-Duclos disease or “dysplastic cerebellar gangliocytoma” (OMIM 158350) is a rare disorder of the cerebellum that is usually included into the group of focal cerebellar dysplasia [36]. In contrast to Chudley-McCullough syndrome, α-dystroglycanopathies, *GPR56*-related polymicrogyria, and Poretti-Boltshauser syndrome, in Lhermitte-Duclos disease the orientation of the cerebellar foliae is preserved, while the volume of multiple cerebellar foliae is diffusely increased [37]. This results in a characteristic neuroimaging pattern of localized lamellar hypertrophy: the inner portions of the folia are T1 hypointense and T2 hyperintense, while the outer portions are T1 isointense and T2 iso- to hypointense.

### Cerebellar dysmorphia

Cerebellar dysmorphia is a term that we recently coined to refer to peculiar morphological anomalies that we saw in few patients with neurofibromatosis type 1 [38]. Cerebellar dysmorphia refers to enlargement of a cerebellar hemisphere with widening of the interfolial spaces of its posterior part, which is bulky (like an appendicular portion of additional cerebellar tissue) and crosses the midline (Fig. 1j-k).

### Cerebellar atrophy

Cerebellar atrophy is a relatively common neuroimaging finding in pediatric neurology and neuroradiology. Cerebellar atrophy is defined as a cerebellum with initially normal structures, in a posterior fossa with normal size, which displays enlarged fissures (interfolial spaces) in comparison to the foliae secondary to loss of tissue (Fig. 1l) [1]. Cerebellar atrophy implies irreversible loss of tissue and result from an ongoing progressive disease until a final stage is reached or a single injury, e.g. an intoxication or infectious event.

Cerebellar atrophy is a non-specific neuroimaging finding and has been associated with a long list of pediatric diseases including genetic and acquired causes. We proposed a pattern recognition approach for hereditary pediatric cerebellar atrophy [1]. We differentiated between isolated (“pure”) cerebellar atrophy and cerebellar atrophy associated (“plus”) with additional neuroimaging findings including hypomyelination, progressive white matter abnormalities, signal change of the dentate nucleus, cerebellar cortex T2-hyperintensity, and basal ganglia involvement.

The distinction between cerebellar atrophy and cerebellar hypoplasia is not difficult in theory, but can be problematic or impossible in practice based on a single examination. In children with non-progressive cerebellar ataxia (*i.e.* with an obviously longstanding static situation), enlarged cerebellar sulci mimicking cerebellar atrophy CA may be seen and are stable over time (stable follow-up up to 20 years) [12–14]. In this situation, we prefer the term cerebellar hypoplasia because of the non-progressive course of clinical and neuroimaging findings and favor a malformative instead of a degenerative pathomechanism. In addition, cerebellar atrophy may be superimposed to cerebellar hypoplasia as shown in some forms of pontocerebellar hypoplasia and some children with congenital disorder of glycosylation type 1a [21, 24].

## Conclusion

**Fig. 2:**
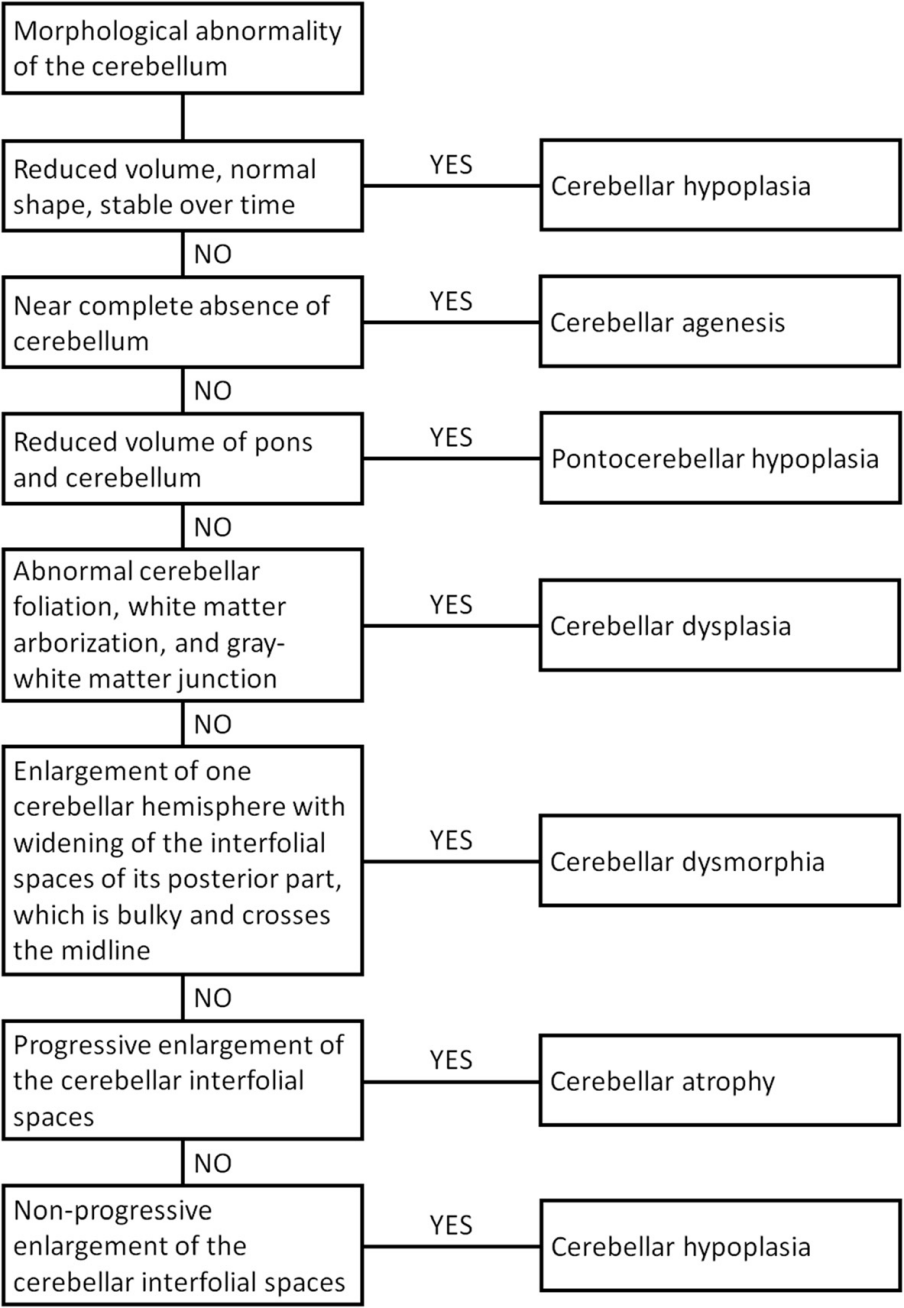
Flow chart developed for reviewing MRI studies displaying morphological abnormalities of the cerebellum

Neuroimaging enables the distinction between various morphological pattern of cerebellar anomalies including cerebellar agenesis, cerebellar hypoplasia, pontocerebellar hypoplasia, cerebellar dysplasia, cerebellar dysmorphia, and cerebellar atrophy (Fig. 2). For these patterns, different diagnostic criteria based on neuroimaging findings have been delineated. Familiarity with these diagnostic criteria is mandatory for a correct diagnosis and a targeted work-up to avoid unnecessary investigations. A correct diagnosis is essential for early therapy, prognosis, and counseling of the affected children and their family.
